# Midwives’ Knowledge and Attitudes Regarding Perinatal Care for Women With Disabilities

**DOI:** 10.7759/cureus.67456

**Published:** 2024-08-22

**Authors:** Athina Diamanti, Maria Vasiliki Zampeli, Chrysoula Taskou, Aikaterini Lykeridou, Antigoni Sarantaki

**Affiliations:** 1 Department of Midwifery, University of West Attica, Athens, GRC

**Keywords:** disability-inclusive training, healthcare barriers, midwives' attitudes, women with disabilities, perinatal care

## Abstract

Introduction: Women with disabilities (WWD) face significant barriers to accessing quality perinatal care, resulting in adverse outcomes for mothers and newborns. Midwives are crucial in providing this care, but their knowledge and attitudes can impact the quality of services delivered. This study aims to examine midwives' knowledge and attitudes toward perinatal care for WWD and identify factors influencing these aspects.

Methods: A cross-sectional study was conducted from January to April 2023, involving 149 midwives from various healthcare settings. Data were collected using a self-administered questionnaire addressing demographics, knowledge, attitudes, and perceived barriers related to perinatal care for WWD. Descriptive statistics and non-parametric tests were used for analysis, with a significance level set at 0.05.

Results: The sample comprised predominantly female midwives (146, 98%), with a mean age of 33.7 years and a mean of 9.8 years of work experience. Only 48 (32.2%) reported workplaces equipped for perinatal care for WWD. Most participants rated the perinatal care services in Greece for WWD as moderate (87, 58.4%) and believed that the medical staff's knowledge in this area was insufficient (148, 99.3%). The mean knowledge score was 35 points out of 100, indicating a low level of knowledge. Key barriers included the lack of adapted services (148, 99.3%) and insufficient infrastructure (143, 96%). Despite these challenges, 142 (95.3%) midwives supported the right of WWD to have children. Significant correlations were found between higher knowledge scores and less specialized attitudes, while more perceived barriers correlated with a greater need for further education.

Conclusions: This study highlights the urgent need for improved education and training programs for midwives to enhance their knowledge and attitudes toward perinatal care for WWD. Addressing educational and structural barriers is essential to provide equitable and high-quality care. Policymakers should prioritize creating inclusive healthcare environments and support ongoing professional development for midwives.

## Introduction

The most accepted definition of disability is that provided by the WHO through the International Classification of Functioning, Disability, and Health (ICF) [1]. According to the ICF, disability is defined as an umbrella term encompassing impairments, activity limitations, and participation restrictions resulting from a combination of health conditions and contextual factors. These contextual factors include external environmental influences such as social behaviors and internal personal factors like gender and social background [1].

Women with disabilities (WWD) often encounter significant barriers to accessing quality healthcare, including perinatal care, which can lead to adverse outcomes for both mothers and newborns [2,3]. These barriers encompass physical inaccessibility, such as the absence of ramps and accessible examination tables, as well as attitudinal challenges, where healthcare providers may harbor biases or lack knowledge about disability-specific needs. Additionally, systemic issues such as insufficient training for healthcare professionals and inadequate healthcare policies further impede access to quality care. Consequently, WWD may experience higher rates of complications during pregnancy and childbirth, negatively affecting their health and the health of their newborns [2,3].

Midwives play a critical role in delivering perinatal care to WWD, and their knowledge and attitudes significantly impact the quality of care provided. However, there is a dearth of research globally examining midwives' knowledge and attitudes toward providing perinatal care for WWD. Most current research focuses on the experiences of disabled women with nursing care rather than exploring the attitudes of healthcare professionals. Several factors can influence midwives' knowledge and attitudes toward perinatal care for WWD. Education and training have a crucial role [4,5].

Midwives who receive disability-inclusive care education and training are more likely to have a positive attitude towards this population and provide appropriate care [6,7]. Conversely, midwives lacking such training may hold misconceptions and negative attitudes, leading to suboptimal care. In addition, the lack of resources and support may impact midwives' ability to provide appropriate care to WWD. Limited access to facilities, insufficient equipment, and inadequate time can hinder their ability to deliver optimal care. Furthermore, a lack of support from colleagues and superiors may negatively impact their confidence and willingness to care for WWD [6,8].

Midwives' attitudes and beliefs toward disability can also significantly impact care provision. Those holding ableist attitudes may view disability as a personal tragedy or medical problem, leading to a deficit-based approach to care [8]. Conversely, midwives adopting a social model of disability may see it as a natural part of human diversity and prioritize equitable and inclusive care [9]. Communication barriers between midwives and WWD can also impact care quality. Midwives may struggle to effectively communicate with women who have different communication needs, such as those who are deaf or have intellectual disabilities, leading to misunderstandings, miscommunication, and inadequate care [5].

This study aims to advance knowledge in perinatal care and promote ethical research practices in midwifery. The results of this study will provide valuable information on midwives' knowledge and attitudes, enabling the development of strategies to improve the quality of perinatal care and identify barriers that limit optimal treatment and care for WWD.

## Materials and methods

Study population and setting

This cross-sectional study was conducted from January to April 2023. The study targeted a purposive sample of 149 midwives working in various healthcare settings, including hospitals, birth centers, health centers, and clinics. These settings were chosen to capture a broad spectrum of experiences and attitudes among midwives toward perinatal care for WWD.

Inclusion and exclusion criteria

Participants were included in the study if they were currently practicing midwives within the selected healthcare settings and had direct experience with perinatal care. Exclusion criteria included midwives who were not actively practicing or those without direct patient care responsibilities. The sample size of 149 participants was determined based on the expected prevalence of midwives' engagement with WWD and the need to ensure a statistically significant representation of different attitudes and knowledge levels.

Study design

The study utilized a cross-sectional design, employing a self-administered questionnaire developed through a comprehensive review of existing literature on midwives' knowledge and attitudes toward perinatal care for WWD. The questionnaire included both closed-ended and open-ended questions, designed to gather detailed information on several aspects (see Appendices).

Demographics

Information about age, gender, educational background, years of experience, and current workplace setting of the participants was gathered.

Knowledge Assessment 

Questions aimed at evaluating midwives' knowledge of perinatal care practices specifically for WWD were asked, using a scoring system with a maximum of 100 points.

Attitude Assessment

Midwives' attitudes toward WWD, particularly in terms of their rights to have children and the appropriateness of various delivery methods, were evaluated. Attitude scores were based on a scale from 0 to 7 points.

Perceived Barriers

Barriers to providing optimal perinatal care to WWD, including a lack of infrastructure, insufficient training, and attitudinal challenges, were identified.

Educational Needs

The perceived need for additional training and specialization in caring for WWD was assessed.

Data collection and statistical analysis

Data were collected through the administration of the questionnaire to participants during their work hours. Participation was voluntary, and respondents were assured of the confidentiality of their responses. We used means, standard deviations (SDs), medians, and interquartile ranges to describe the quantitative variables. We used absolute (N) and relative (%) frequencies to describe the qualitative variables. We used the Kolmogorov-Smirnov test to test the normality of the distributions. The non-parametric Mann-Whitney test was used to compare quantitative variables between two groups (Knowledge Score, Barrier Score, Need for Further Training, Knowledge Score for Structures, and Attitude Rating).

We used the non-parametric Kruskal-Wallis test to compare quantitative variables between more than two groups. To control for type I error due to multiple comparisons, the Bonferroni correction was used, in which the significance level was 0.05/k (κ = number of comparisons). Spearman's non-parametric correlation coefficient (rho) was used to test the relationship between two quantitative variables. Correlation is considered low when the correlation coefficient (rho) ranges from 0.1 to 0.3, moderate when the correlation coefficient ranges from 0.31 to 0.5, and high when the coefficient is greater than 0.5. Linear regression analysis, using logarithmic transformations, was used to find independent factors related to specific scores in relation to the perinatal care of WWD, which yielded the coefficients of dependence (βs) and their standard errors (SEs). The internal reliability of the questionnaire was tested using Cronbach's coefficient (α). The significance levels were two-sided, and the statistical significance was set at 0.05. The statistical program IBM SPSS Statistics for Windows, Version 22, (Released 2013; IBM Corp., Armonk, New York, United States) was used for the analysis.

Ethical considerations

The study was conducted in compliance with ethical guidelines, ensuring that all participants were fully informed about the study's purpose, procedures, and their rights as participants. Written informed consent was obtained from all participants, who were assured that they could withdraw from the study at any time without penalty. The confidentiality of all participant data was maintained throughout the study, and the results were reported in a manner that protected the identities of the participants. The study received ethical approval from the relevant committee, with approval number 20/09032022.

## Results

Correlation coefficients between the scores of attitudes, knowledge, barriers, and education in relation to the perinatal care of WWD

A significant negative correlation was found between the knowledge score and the attitude score; therefore, the more the participants' knowledge about people with disabilities and their perinatal care, the less specialized they think their attitude toward them should be during this period (Spearman’s rho: -0.34, p<0.001).

On the contrary, a positive correlation was found between the barrier score and the need for further education; therefore, the more barriers the participants consider existing in providing perinatal care to WWD, the greater the need they feel for further education and expertise in the field (Spearman’s rho: 0.28, p=0.001).

Associations of attitudes, knowledge, barriers, and education in relation to perinatal care of WWD with demographic-work characteristics and corresponding experience/contact with people with disabilities

More knowledge about people with disabilities and their perinatal care is gained by the participants who have performed their professional duties on a person with disabilities (p=0.048), as well as those who do not design a specialized care plan, together with the rest of the professional team, for WWD (p=0.002).

Regarding the score of barriers, more barriers to the provision of perinatal care are seen by the participants who do not have equipment for perinatal care at their workplace for persons with disabilities (p=0.036). Also, the barrier score was found to differ depending on the workplace of the participants, and specifically after the Bonferroni correction, it was found that participants who work in primary health care have a significantly higher barrier score than those who work in secondary or tertiary health care (p =0.046) (Table 1).

**Table 1:** Results for "perinatal care knowledge rating" and "perinatal care barrier rating." +Mann-Whitney, ++Kruskal-Wallis

Question		Knowledge score		p	Barrier rating		p
		Mean value (SD)	Median (interquartile range)		Mean value (SD)	Median (interquartile range)	
Married / cohabitation agreement	No	32.9 (24.4)	40 (20 ─ 60)	0.343 ^+^	7.6 (1.7)	9 (7 ─ 9)	0.614 ^+^
	Yes	37.8 (24.5)	40 (20 ─ 60)		7.6 (1.6)	8 (7 ─ 9)	
Education	School for midwives / bachelor’s degree	36.9 (26.5)	40 (20 ─ 60)	0.430 ^+^	7.4 (1.8)	8 (6 ─ 9)	0.268 ^+^
	Master's degree / doctorate	32.5 (21.5)	40 (20 ─ 40)		7.9 (1.3)	8 (7 ─ 9)	
Workplace and employment	Primary health care	34.3 (23.6)	40 (20 ─ 60)	0.938 ^++^	8.2 (1.4)	9 (7.5 ─ 9)	0.046 ^++^
	Secondary / tertiary health care	35.9 (25.5)	40 (20 ─ 60)		7.4 (1.7)	8 (7 ─ 9)	
	Freelance	32.5 (21.9)	40 (20 ─ 50)		7.8 (1.3)	8 (7 ─ 9)	
Does your workplace have equipment for the perinatal care of disabled individuals?	No	33.1 (24.4)	40 (20 ─ 60)	0.190 ^+^	7.7 (1.6)	9 (7 ─ 9)	0.036 ^+^
	Yes	39.2 (24.4)	40 (20 ─ 60)		7.4 (1.7)	7.5 (7 ─ 9)	
During your work, have you performed your professional duties for a woman with a disability?	No	31.1 (22.7)	40 (20 ─ 40)	0.048 ^+^	7.5 (1.6)	8 (6 ─ 9)	0.260 ^+^
	Yes	38.5 (25.6)	40 (20 ─ 60)		7.7 (1.6)	9 (7 ─ 9)	
Indicate your personal experience or contact with a woman with a disability including professional contact (e.g., colleague, person living in the same building, etc.); how frequent is it?	Never/rarely	36.6 (25.4)	40 (20 ─ 60)	0.169 ^++^	7.7 (1.5)	8 (7 ─ 9)	0.893 ^++^
	Less than once a month	35.2 (20.5)	40 (20 ─ 40)		7.5 (1.9)	8 (7 ─ 9)	
	Weekly/daily	24.2 (24.6)	20 (0 ─ 40)		7.5 (1.6)	8 (6 ─ 9)	
During the prenatal check-up, a pregnant woman with a disability and her companion arrive. Do you decide whom you will address first?	No	36.7 (25.3)	40 (20 ─ 60)	0.150 ^+^	7.6 (1.6)	8 (7 ─ 9)	0.581 ^+^
	Yes	31.1 (22.4)	40 (20 ─ 40)		7.5 (1.6)	8 (6 ─ 9)	
Do you and the rest of the team design a specialized care plan for a woman with a disability?	No	45.5 (22.2)	40 (40 ─ 60)	0.002 ^+^	7.6 (1.8)	8.5 (6.5 ─ 9)	0.971 ^+^
	Yes	31.2 (24.3)	20 (20 ─ 40)		7.6 (1.6)	8 (7 ─ 9)	
Do you or someone in your family have a disability?	Yes, I have a disability / a relative has a disability	40.8 (27.7)	40 (20 ─ 60)	0.108 ^+^	7.9 (1.5)	9 (7 ─ 9)	0.194 ^+^
	No, I don't have a disability and none of my relatives have a disability	32.2 (22.4)	40 (20 ─ 60)		7.5 (1.7)	8 (7 ─ 9)	

A greater need for further training and specialization in the perinatal care of WWD is felt by the participants who have exercised their professional duties in the care of a person with a disability (p=0.011), while there is less need for additional training by those who decide, depending on the case, whom they will turn to first when they come within the framework of prenatal check-up: a pregnant woman with disabilities or her companion (p=0.046).

Participants who are married or cohabiting, who have equipment at their workplace, and who have exercised their professional duties have more knowledge about structures and services for the provision of perinatal care for people with disabilities (p=0.048 and p=0.001, respectively). In addition, those who design a specialized care plan, together with the rest of the professional team, for the disabled woman and those participants who either themselves have or have a relative who has some disability have a higher knowledge score about the structures (p=0.001 and p=0.034, respectively).

Also, the knowledge score for the structures was found to differ depending on how frequent their personal experience or contact is with a person with a disability, and specifically after the Bonferroni correction, it was found that the participants who have personal experience/contact with a person with a disability weekly or daily have a significantly higher knowledge score than those who have never or rarely experienced this (p=0.018) (Table 2).

**Table 2:** Results for "score of need for further training and specialization in perinatal care for PWD" and "score of knowledge for PWD perinatal care structures." +Mann-Whitney, ++Kruskal-Wallis PWD: persons with disabilities

Question		Need rating for further education		p	Knowledge score for structures		p
		Mean value (SD)	Median (interquartile range)		Mean value (SD)	Median (interquartile range)	
Married / cohabitation agreement	No	1.8 (0.5)	2 (2 ─ 2)	0.496 ^+^	0.7 (0.8)	1 (0 ─ 1)	0.024 ^+^
	Yes	1.8 (0.4)	2 (2 ─ 2)		1.2 (1.3)	1 (0 ─ 2)	
Education	School for midwives / bachelor’s degree	1.8 (0.4)	2 (2 ─ 2)	0.684 ^+^	1 (1,1)	1 (0 ─ 2)	0.814 ^+^
	Master's degree / doctorate	1.8 (0.5)	2 (2 ─ 2)		0.9 (1)	1 (0 ─ 1.5)	
Workplace and employment	Primary health care	1.9 (0.4)	2 (2 ─ 2)	0.600 ^++^	1.1 (1.2)	1 (0 ─ 2)	0.293 ^++^
	Secondary / tertiary health care	1.8 (0.5)	2 (2 ─ 2)		0.9 (1.1)	1 (0 ─ 1)	
	Freelance	1.9 (0.3)	2 (2 ─ 2)		1 (0.8)	1 (0 ─ 1.5)	
Does your workplace have equipment for the perinatal care of disabled individuals?	No	1.8 (0.4)	2 (2 ─ 2)	0.777 ^+^	0.8 (0.9)	1 (0 ─ 1)	0.048 ^+^
	Yes	1.8 (0.5)	2 (2 ─ 2)		1.2 (1.2)	1 (0 ─ 2)	
During your work, have you performed your professional duties for a woman with a disability?	No	1.7 (0.5)	2 (1 ─ 2)	0.011 ^+^	0.6 (0.8)	0 (0 ─ 1)	0.001 ^+^
	Yes	1.9 (0.3)	2 (2 ─ 2)		1.2 (1.2)	1 (0 ─ 2)	
Indicate your personal experience or contact with a woman with disability including professional contact (e.g., colleague, person living in the same building, etc.); how frequent is it?	Never/rarely	1.8 (0.5)	2 (2 ─ 2)	.305 ^++^	0.8 (1.1)	0 (0 ─ 1)	0.018 ^++^
	Less than once a month	1.8 (0.5)	2 (2 ─ 2)		1.1 (1)	1 (0 ─ 2)	
	Weekly/daily	1.9 (0.2)	2 (2 ─ 2)		1.4 (1.1)	1 (1 ─ 2)	
During the prenatal check-up, a pregnant woman with a disability and her companion arrive. Do you decide whom you will address first?	No	1.8 (0.4)	2 (2 ─ 2)	0.046 ^+^	0.9 (1)	1 (0 ─ 1)	0.587 ^+^
	Yes	1.7 (0.5)	2 (1 ─ 2)		1 (1,1)	1 (0 ─ 2)	
Do you and the rest of the team design a specialized care plan for a woman with a disability?	No	1.9 (0.4)	2 (2 ─ 2)	0.594 ^+^	0.5 (0.9)	0 (0 ─ 1)	0.001 ^+^
	Yes	1.8 (0.5)	2 (2 ─ 2)		1.1 (1.1)	1 (0 ─ 2)	
Do you or someone in your family have a disability?	Yes, I have a disability / a relative has a disability	1.9 (0.3)	2 (2 ─ 2)	0.222 ^+^	1.2 (1.3)	1 (0 ─ 2)	0.034 ^+^
	No, I don't have a disability and none of my relatives have a disability	1.8 (0.5)	2 (2 ─ 2)		0.8 (0.9)	1 (0 ─ 1)	

The participants who plan a specialized care plan, together with the rest of the professional team, for the disabled woman have a more specialized attitude towards providing perinatal care to a disabled woman (p=0.010).

Also, the attitude score was found to differ depending on the workplace of the participants, and specifically after the Bonferroni correction, it was found that the participants who work in primary health care have a more specialized attitude toward the provision of perinatal care to WWD, compared to those who work in secondary or tertiary health care (p =0.050) (Table 3).

**Table 3:** Results for the "rating of attitudes in the provision of perinatal care for WWDs." +Mann-Whitney, ++Kruskal-Wallis

Question		Attitude rating		P
		Mean value (SD)	Median (interquartile range)	
Married / cohabitation agreement	No	4.3 (1.7)	4 (3 ─ 5)	0.813 ^+^
	Yes	4.4 (1.5)	4 (3 ─ 5)	
Education	School for midwives / bachelor’s degree	4.2 (1.7)	4 (3 ─ 5)	0.407 ^+^
	Master's degree / doctorate	4.5 (1.5)	5 (3 ─ 5)	
Workplace and employment	Primary health care	4.8 (1.6)	5 (3.5 ─ 6)	0.050 ^++^
	Secondary / tertiary health care	4.2 (1.6)	4 (3 ─ 5)	
	Freelance	4.5 (1.7)	4.5 (3.5 ─ 5)	
Does your workplace have equipment for the perinatal care of disabled individuals?	No	4.4 (1.6)	5 (3 ─ 5)	0.318 ^+^
	Yes	4.2 (1.6)	4 (3 ─ 5)	
During your work, have you performed your professional duties for a woman with a disability?	No	4.2 (1.4)	4 (3 ─ 5)	0.357 ^+^
	Yes	4.4 (1.7)	5 (3 ─ 6)	
Indicate your personal experience or contact with a woman with a disability including professional contact (e.g., colleague, person living in the same building, etc.); how frequent is it?	Never/rarely	4.2 (1.7)	4 (3 ─ 5)	0.136 ^++^
	Less than once a month	4.2 (1.4)	4 (3 ─ 5)	
	Weekly/daily	4.9 (1.6)	5 (4 ─ 6)	
During the prenatal check-up, a pregnant woman with a disability and her companion arrive. Do you decide whom you will address first?	No	4.3 (1.6)	4 (3 ─ 5)	0.470 ^+^
	Yes	4.5 (1.5)	5 (3 ─ 5)	
Do you and the rest of the team design a specialized care plan for a woman with a disability?	No	3.8 (1.7)	3.5 (2.5 ─ 5)	0.010 ^+^
	Yes	4.5 (1.5)	5 (4 ─ 6)	
Do you or someone in your family have a disability?	Yes, I have a disability / yes, a relative has a disability	4.3 (1.6)	4 (3 ─ 5)	0.844 ^+^
	No, I do not have a disability and none of my relatives have a disability	4.3 (1.6)	4 (3 ─ 5.5)	

The older the participants, the fewer barriers they see in providing perinatal care to a woman with disabilities (Spearman’s rho -0.19, p=0.023). Also, the older they are and the more years of service they have, the more knowledge they have about structures and services for providing perinatal care to people with disabilities (Spearman’s rho -0.26, p=0.001 and Spearman’s rho 0.22, p=0.006, respectively).

Then, multivariate linear regressions were performed with dependent variables as the scores of attitudes, knowledge, barriers, and education in relation to the perinatal care of WWD and independent variables as the demographic-work characteristics of the participants and their respective experience/contact with people with disabilities. Analyses were performed using logarithmic transformations. 

The score of knowledge of perinatal care for WWD was found to be independently related only to whether a specialized care plan is planned for a woman with disabilities, and specifically was a lower score when such a plan was formed; therefore, those who plan a specialized plan, together with the rest of the professional group, for the perinatal care of a woman with disabilities have less knowledge (p=0.021).

The score of barriers in the perinatal care of the WWD was found to be independently associated with participants' age and workplace. Specifically, the older the participants, the fewer barriers they see in providing perinatal care to a woman with disabilities (p=0.035). Participants who work in secondary or tertiary health care have a significantly lower barrier score; therefore, they see fewer barriers in providing perinatal care to a woman with disabilities, compared to those who work in primary health care (p=0.032).

The score of need for further education and specialization in perinatal care of WWD was found to be independently related only to participants' professional experience/contact with a person with a disability; specifically, the participants who have had to exercise their professional duties with a disabled person feel a greater need for further training and specialization in perinatal care for disabled women (p=0.025) (Table 4).

**Table 4:** Multivariate linear regressions with dependent variables "knowledge rating %," "barrier rating," and "need rating for further education." +dependence coefficient, ++standard error of coefficient

Parameter		Knowledge score		Barrier rating		Need rating for further education	
		β ^+ ^( SE ^++ ^)	P	β ^+ ^( SE ^++ ^)	P	β ^+ ^( SE ^++ ^)	P
Age (years)		0.003 (0.019)	0.866	-0.006 (0.003)	0.035	- 0.002 (0.004)	0.229
Years of service		-0.010 (0.019)	0.614	0.004 (0.003)	0.166	- 0.005 (0.003)	0.078
Married / cohabitation agreement	No (report)		P	β ^+ ^( SE ^++ ^)	P	β ^+ ^( SE ^++ ^)	P
	Yes	0.241 (0.141)	0.090	0.028 (0.021)	0.185	0.011 (0.020)	0.585
Education	School for midwives / bachelor’s degree (reference)						
	Master's degree / docorate	-0.037 (0.111)	0.737	0.030 (0.016)	0.069	-0.014 (0.016)	0.362
Workplace and employment	Primary health care (reference)			β ^+ ^( SE ^++ ^)	P	β ^+ ^( SE ^++ ^)	P
	Secondary / tertiary health care	0.001 (0.137)	0.999	-0.043 (0.020)	0.032	-0.013 (0.019)	0.486
	Freelance	-0.010 (0.180)	0.957	-0.034 (0.026)	0.198	0.005 (0.025)	0.844
Does your workplace have equipment for the perinatal care of disabled individuals?	No (report)		P	β ^+ ^( SE ^++ ^)	P	β ^+ ^( SE ^++ ^)	P
	Yes	0.147 (0.116)	0.210	-0.026 (0.017)	0.125	-0.007 (0.016)	0.655
During your work, have you performed your professional duties for a woman with a disability?	No (report)		P	β ^+ ^( SE ^++ ^)	P	β ^+ ^( SE ^++ ^)	P
	Yes	0.153 (0.116)	0.189	0.021 (0.017)	0.212	0.037 (0.016)	0.025
Indicate your personal experience or contact with a woman with disability including professional contact (e.g., colleague, person living in the same building, etc.); how frequent is it?	Never/rarely (report)			β ^+ ^( SE ^++ ^)	P	β ^+ ^( SE ^++ ^)	P
	Less than once a month	0.009 (0.142)	0.952	-0.023 (0.021)	0.266	-0.014 (0.020)	0.498
	Weekly/daily	0.023 (0.012 )	0, 18 8	-0.030 (0.025)	0.240	0.019 (0.024)	0.423
During the prenatal check-up, a pregnant woman with a disability and her companion arrive. Do you decide whom to address first?	No (report)		P	β ^+ ^( SE ^++ ^)	P	β ^+ ^( SE ^++ ^)	P
	Yes	-0.051 (0.119)	0.672	0.010 (0.017)	0.579	-0.018 (0.017)	0.295
Do you and the rest of the team design a specialized care plan for a woman with a disability?	No (report)		P	β ^+ ^( SE ^++ ^)	P	β ^+ ^( SE ^++ ^)	P
	Yes	-0.290 (0.124)	0.021	0.014 (0.018)	0.440	-0.016 (0.017)	0.354
Do you or someone in your family have a disability?	No, I do not have a disability and none of my relatives have a disability (reference)			β ^+ ^( SE ^++ ^)	P	β ^+ ^( SE ^++ ^)	P
	Yes, I have a disability / a relative of mine has a disability	0.173 (0.118)	0.144	0.027 (0.017)	0.122	0.023 (0.017)	0.177

WWD perinatal care structures score was found to be independently related to the age and years of service of the participants, to whether there is appropriate equipment at their workplace, to professional experience/contact with a WWD, to whether a specialized care plan for a woman with disabilities is there, and whether they or any of their relatives have a disability.

Specifically, the older the participants, the more knowledge they have about the structures and services related to the provision of perinatal care to WWD (p=0.001). Also, the more years of service they have, the more knowledge they have about structures and services related to the provision of perinatal care to WWD (p=0.005). Those who have equipment in their workplace for the perinatal care of these individuals have more knowledge related to the structures for perinatal care of WWD (p=0.031). Those who have exercised their professional duties during their work with a disabled person also have a higher score of knowledge about the structures (p=0.002). In addition, those who design a specialized care plan, together with the rest of the professional team, have more knowledge about the structures when it comes to a disabled woman (p=0.001). Finally, those participants who either themselves have or have a relative who has some disability have a significantly higher knowledge score about the structures (p=0.017).

The score of attitudes in the provision of perinatal care for WWD was found to be independently related only to whether a specialized perinatal care plan is planned for disabled women. In particular, the higher the score, the more specialized the attitude toward the perinatal care of disabled women of the participants who, when necessary, together with the rest of the professional team, plan a specialized plan for the care of WWD (p=0.013) (Table 5).

**Table 5:** Multivariate linear regressions with dependent variables "score of knowledge for structures" and "score of attitudes." +dependence coefficient, ++standard error of coefficient

Parameter		Knowledge score for structures		Attitude rating	
		β ^+ ^( SE ^++ ^)	P	β ^+ ^( SE ^++ ^)	P
Age (years)		0.020 (0.006)	0.001	0.001 (0.005)	0.842
Years of service		0.017 (0.006)	0.005	0.001 (0.005)	0.902
Married / cohabitation agreement	No (report)			β ^+ ^( SE ^++ ^)	P
	Yes	0.017 (0.043)	0.697	-0.002 (0.035)	0.959
Education	School for midwives / bachelor’s degree (reference)			β ^+ ^( SE ^++ ^)	P
	Master's degree / doctorate	0.020 (0.034)	0.562	0.036 (0.027)	0.190
Workplace and employment	Primary health care (reference)			β ^+ ^( SE ^++ ^)	P
	Secondary / tertiary health care	-0.043 (0.042)	0.305	-0.051 (0.033)	0.130
	Freelance	0.016 (0.055)	0.767	-0.040 (0.044)	0.360
Does your workplace have equipment for the perinatal care of disabled individuals?	No (report)			β ^+ ^( SE ^++ ^)	P
	Yes	0.077 (0.036)	0.031	-0.008 (0.028)	0.769
During your work, have you performed your professional duties for a woman with a disability?	No (report)			β ^+ ^( SE ^++ ^)	P
	Yes	0.115 (0.035)	0.002	0.009 (0.028)	0.761
Indicate your personal experience or contact with a woman with a disability including professional contact (e.g., colleague, person living in the same building, etc.); how frequent is it?	Never/rarely (report)			β ^+ ^( SE ^++ ^)	P
	Less than once a month	0.047 (0.043)	0.276	0.002 (0.035)	0.944
	Weekly/daily	0.036 (0.052)	0.496	0.026 (0.042)	0.530
During the prenatal check-up, a pregnant woman with a disability and her companion arrive. Do you decide whom you will address first?	No (report)			β ^+ ^( SE ^++ ^)	P
	Yes	0.001 (0.036)	0.980	0.025 (0.029)	0.391
Do you and the rest of the team design a specialized care plan for a woman with a disability?	No (report)			β ^+ ^( SE ^++ ^)	P
	Yes	0.131 (0.038)	0.001	0.076 (0.030)	0.013
Do you or someone in your family have a disability?	No, I do not have a disability and none of my relatives have a disability (reference)			β ^+ ^( SE ^++ ^)	P
	Yes, I have a disability / a relative of mine has a disability	0.087 (0.036)	0.017	-0.001 (0.029)	0.966

## Discussion

Workplace preparedness and knowledge gaps

Only 48 (32.2%) midwives reported having workplaces equipped for the perinatal care of individuals with disabilities. The evaluation of maternity care services for WWD in Greece showed that 87 (58.4%) participants rated the services as moderate, and 148 (99.3%) felt the medical staff lacked sufficient knowledge of and specialization in perinatal care for WWD. A significant portion, (68, 45.6%), experienced fear/anxiety when providing perinatal care to WWD, while 117 (78.5%) believed showing pity/sympathy should be avoided. Moreover, 142 (95.3%) participants supported the right of WWD to have children, with preferences for delivery methods varying based on the type of disability.

Barriers and attitudes

Midwives' knowledge about perinatal care for WWD was generally low. Key barriers included the lack of adapted services (148, 99.3%) and insufficient infrastructure (143, 96%). There was a strong consensus (144, 96.6%) on the need for additional training in perinatal care for WWD, with a mean need rating of 1.8 points. Attitudes toward perinatal care for WWD showed a strong preference for prioritizing these individuals in care provision (133, 89.3%) and the need for specialized parenting courses (124, 83.2%). Significant correlations indicated that higher knowledge scores were associated with less specialized attitudes, while more perceived barriers correlated with a greater need for further education. Factors such as age, years of service, and having equipment for perinatal care at the workplace significantly influenced knowledge and attitudes toward WWD.

International comparisons and insights

In Eswatini, midwives face significant challenges when providing maternity care to women with mobility disabilities. The study by Temane et al. found that midwives experience both physical and emotional strain due to the lack of appropriate equipment and infrastructure. They reported frustration over the absence of special beds, examination tables, and accessible facilities, which hindered their ability to deliver holistic and competent care. Additionally, the midwives highlighted the need for specific guidelines and protocols to better address the unique needs of women with mobility disabilities. Despite these challenges, there is a call for improved training and resources to support midwives in providing adequate care for this population [10].

In another study from Eswatini, midwives' experiences highlighted a troubling pattern of unprofessional and abusive behavior toward WWD. Participants in the study reported instances of physical and emotional abuse by midwives, stemming from a lack of understanding and sensitivity toward disabled women's needs. The study underscored the critical need for professional training to instill empathy, patience, and appropriate care techniques in midwives. Additionally, it pointed out the importance of developing supportive infrastructure and protocols to ensure that these women receive respectful and effective maternity care [11].

The study by Redshaw et al., which was conducted among Polish midwives, revealed a significant gap in the preparedness to care for WWD during pregnancy, childbirth, and the postpartum period. Most midwives reported a lack of established standards and training, which led to increased anxiety and psychological burden when caring for disabled women. The survey indicated that many midwives felt their workplaces were not adequately adjusted to cater to the needs of disabled women, which compounded their stress and uncertainty in providing care. The findings emphasized the urgent need for the development of specific training programs and standards to improve midwifery care for WWD [12].

Health professionals in Austria expressed similar concerns regarding their ability to provide adequate maternity care to WWD. They pointed out the lack of proper training and experience as significant barriers to effective care. Moreover, the absence of clear guidelines and collaborative practices often led to inconsistent and suboptimal care for disabled women. The study emphasized the need for comprehensive training programs that focus on disabilities and the implementation of standardized protocols to ensure that all health professionals can deliver consistent and competent care [13].

The study by Devkota et al. found that healthcare providers in rural Nepal generally hold negative attitudes toward WWD. This attitude is reflected in their overall low scores on the Attitude Toward Disabled Persons (ATDP) Scale, with nurse-midwives obtaining the highest scores and female community health volunteers (FCHVs) the lowest. Younger providers and those working in urban areas showed more positive attitudes compared to older and rural-based providers. Interestingly, prior exposure to providing care for WWD and receiving disability training did not significantly improve the providers' attitudes [14].

The study by Smeltzer et al. highlighted significant gaps in formal training for clinicians regarding disability care, with none of the interviewed clinicians having received specific education on this topic during their medical training. Despite entering this field incidentally, clinicians found caring for women with physical disabilities to be highly rewarding, describing it as both a moral duty and a professional fulfillment. However, they faced systemic barriers such as inadequate information, poor continuity of care, and inaccessible facilities, which hindered their ability to provide optimal care. The clinicians often took on the role of educators to share their experiences and improve peer knowledge and sensitivity toward disability care. The study concluded with a call for integrating comprehensive disability care training into medical education to better prepare clinicians for the growing number of pregnant WWD [15].

The role of digital tools in enhancing care

In recent years, web-based platforms have emerged as vital resources for disseminating health-related information to both healthcare professionals and women of reproductive age. These platforms offer accessible, evidence-based content that can enhance knowledge and inform decision-making processes. For example, the development of the "Reproductive Life Plan" website in Sweden demonstrated how targeted online tools could effectively increase fertility awareness and guide pre-conception health practices among users​ [16]. Similarly, a study among Bulgarian women highlighted the demand for a web-based educational platform as a primary source of pre-conception health information, emphasizing its potential to improve awareness and attitudes toward pre-conception care​ [17]. Another innovative platform developed in Italy provided evidence-based recommendations through videos, which were particularly engaging for various topics like nutrition and breastfeeding, demonstrating the adaptability of digital content to user preferences​ [18]. These platforms not only serve as educational tools for the public but also as valuable resources for healthcare providers, enabling them to offer more informed and personalized care. Integrating such digital tools into healthcare practices could bridge existing knowledge gaps and support the delivery of high-quality, inclusive care.

Strengths of the study

This study makes a significant contribution to the existing literature by providing empirical data on midwives' knowledge and attitudes toward perinatal care for WWD in Greece, highlighting critical barriers such as inadequate infrastructure and insufficient services, and identifying correlations between knowledge levels and attitudes toward specialized care. These findings underscore the urgent need for comprehensive, disability-inclusive education and training programs for midwives, improvements in healthcare infrastructure, and the development of inclusive healthcare policies. The study's results offer a strong foundation for future research and policy interventions aimed at enhancing the quality of perinatal care for WWD.

Limitations

Several limitations should be considered when interpreting the results of this study. The use of self-administered questionnaires may introduce response bias, as participants might provide socially desirable answers. Additionally, the study's cross-sectional design limits the ability to draw causal inferences. Future research should employ longitudinal methods to better understand the evolution of midwives' knowledge and attitudes over time. Furthermore, expanding the sample size and including midwives from diverse geographic and socioeconomic backgrounds would enhance the generalizability of the findings.

## Conclusions

This study underscores the urgent need for enhanced education and training programs for midwives to improve their knowledge and attitudes toward perinatal care for WWD. To improve perinatal care for WWD, it is crucial to implement comprehensive disability-inclusive training programs for midwives, integrated into both undergraduate education and ongoing professional development. Enhancing healthcare infrastructure with accessible equipment and developing inclusive healthcare policies that mandate specialized services and support systems are essential steps. Promoting interdisciplinary collaboration among healthcare professionals ensures holistic care, while ongoing research and data collection are necessary to identify gaps and assess the effectiveness of implemented strategies. By prioritizing these actions, policymakers and healthcare administrators can create an inclusive healthcare environment that provides equitable and high-quality care for all women during pregnancy and childbirth.

## Appendices

Knowledge and attitudes of health professionals in relation to perinatal care of women with disabilities

Questionnaire

Section 1: Demographics

Questions

1) Sex:

a) Male

b) Female

2) Age (years)................

3) Marital status:

a) Single

b) Married

c) Civil Partnership

d) Divorced

4) Education:

a) Midwifery Schools

b) Bachelor’s degree

c) Master’s degree (MSc)

d) Doctorate (PhD)

5) Previous experience in the field of health (total: years and months):

Years: ................................. Months: ........................

6) Workplace and employment:

□ Primary health care

□ Public (Specify exactly where)

…………………………………………………

□ Private practice (Specify exactly where)

…………………………………………………

□ Non-governmental organization (NGO)

□ Secondary health care

□ Public (Specify exactly where)

…………………………………………………

□ Private clinic (Specify exactly where)

…………………………………………………

□ Tertiary health care

□ Public (Specify exactly where)

…………………………………………………

□ Private Hospital (Specify exactly where)

…………………………………………………

□ Freelancer

7. Does your workplace have equipment for the perinatal care of disabled individuals?

a) YES              b) NO

Section 2: Evaluation of midwife care services in women with disabilities

Please choose the correct answers.

Questions

1. How do you evaluate the maternity care services in Greece for individuals with disabilities?

a) Not good at all

b) Average

c) Good

d) Very Good

e) Excellent

2. Do you consider that the knowledge and specialization of the medical and nursing staff on perinatal care for individuals with disabilities are sufficient?

a) No

b) Yes

3. How do you evaluate your level of knowledge regarding perinatal care issues for women with disabilities?

a) I don't know

b) I know moderately

c) I know well

d) I know very well

e) I know excellently

4. Do you feel that you have enough knowledge to use the correct terminology and words that will not contain elements of stigma and discrimination?

a No

b) Yes

Section 3: Data pertaining to the emotions experienced by the participants during the provision of perinatal care to women with disabilities, as well as the emotions that participants believe the women themselves experience during their perinatal care

Please choose the correct answers.

Questions

1. Do you feel comfortable gathering information from the woman about the nature of her disability?

a) No

b) Yes

2. What are your feelings when providing perinatal care to women with disabilities?

a) Fear, anxiety

b) Uncertainty, avoidance, and referral to another healthcare professional

c) Neutral/indifferent

d) Emotional burden (pity, sympathy)

e) Confidence/trust

f) Rejection

3. Which of the following do you consider should be avoided in the care of these women?

a) Expressing your personal opinion on matters concerning them

b) Showing them pity/sympathy

c) Not giving them the opportunity to decide for themselves and participate in their care, as well as in the care of their newborn

4. Which of the following, in your opinion, is the most common emotion experienced by women with disabilities during their perinatal care?

a) Fear for the health of their child

b) Concern about whether they will manage to carry the pregnancy to term

c) Fear of being separated from their child

d) Insecurity about the quality of care they receive due to the lack of knowledge and specialization of healthcare professionals regarding their specific problem

e) Insecurity about the adequacy and quality of parental care they will provide to their child themselves

f) Feeling of loss of control

g) Anxiety that the healthcare professional disapproves of their decision to be pregnant (stigma)

Section 4: Participants’ views regarding the right of women with disabilities to have children as well as the preferred method of childbirth depending on the situation

Please choose the correct answers.

Questions

1. Do you believe in the right of women with disabilities to have children?

a) No

b) Yes

2. What do you consider to be the most appropriate method of delivery for women with disabilities?

For a pregnant woman with mobility impairments (e.g., paraplegic)

a)Vaginal birth

b) Cesarian section

For a pregnant woman with intellectual disabilities (e.g., Down syndrome)

a) Vaginal birth

b) Cesarian section

For a pregnant woman with psychiatric problems (e.g., schizophrenia)

a) Vaginal birth

b) Cesarian section

For a pregnant woman with sensory problems (e.g., vision, hearing

a) Vaginal birth

b) Cesarian section

Section 5: Data concerning the experience/contact of participants with women with disabilities both at a professional level through their perinatal care and at a personal level

Please choose the correct answers.

Questions

1. During your work, have you performed your professional duties for a woman with a disability?

a) No

b) Yes

If yes, how many times did it happen?

Specifically, how many times in the last year?

If yes, during which of the following periods of perinatal care did you provide services to a woman with a disability?

a) Pregnancy

b) Childbirth

c) Postpartum

2. During the prenatal check-up, a pregnant woman with a disability and her companion arrives. Whom do you address first?

a) The woman

b) Her companion

c) Both simultaneously

d) Depending on the situation

3. Do you and the rest of the team design a specialized care plan for a woman with a disability?

a) No

b) Yes

4. Indicate your personal experience or contact with a woman with a disability, including professional contact (e.g., colleague, person living in the same building, etc.); how frequent is it?

a) Never

b) Rarely

c) Less than once a month

d) Weekly

e) Daily

5. Do you or someone in your family have a disability?

a) Yes, I have a disability

b) Yes, a relative of mine has a disability

c) No, I do not have a disability

d) No, no relative of mine has a disability

e) No, I do not have a disability, and no relative of mine has a disability

Section 6: Knowledge of the participants about individuals with disabilities and their perinatal care

Please choose the correct answer.

Questions

1. Which of the following categories do you believe belong to people with disabilities?

a) Mobility impairments

b) Sensory impairments

c) Intellectual impairments

d) Psychiatric impairments

e) Chronic conditions

f) All of the above

2. What do you believe is the percentage of the Greek population that belongs to individuals with disabilities?

a) ˂5%

b) 5-10%

c) 10-20%

3. Do you believe that women with disabilities require more frequent perinatal care?

a) No

b) Yes

4. Do you consider the presence of a neonatologist mandatory/necessary at the birth of a woman with disabilities?

a) No

b) Yes

5. Do you consider a pregnant woman with disabilities to be a high-risk pregnancy?

a) No

b) Yes

Section 7: Factors that constitute potential barriers to better perinatal care for women with disabilities

Please choose the correct answer.

Do you believe that the following constitute barriers to better perinatal care for women with disabilities?

1. The knowledge of healthcare professionals regarding perinatal care for individuals with disabilities

a) No

b) Yes

2. The experience of healthcare professionals regarding perinatal care for individuals with disabilities

a) No

b) Yes

3. Accessibility issues and physical barriers at maternity hospitals and examination centers for individuals with disabilities

a) No

b) Yes

4. The lack of or difficult transportation for individuals with disabilities to maternity hospitals and examination centers

a) No

b) Yes

5. The lack of infrastructure and availability of equipment at maternity hospitals and examination centers (e.g., mammography equipment that does not require the person to stand)

a) No

b) Yes

6. Communication barriers between healthcare professionals and individuals with disabilities (e.g., professionals knowledgeable in sign language)

a) No

b) Yes

7. The lack of services specifically adapted to the needs of individuals with disabilities

a) No

b) Yes

8. The behavior of healthcare professionals toward individuals with disabilities

a) No

b) Yes

9. Stereotypes and stigmatization of individuals with disabilities by healthcare professionals

a) No

b) Yes

Section 8: Need for education on perinatal care in women with disabilities

Please choose the correct answer.

Question

1. Do you consider additional training, specialization, and lifelong learning for ALL medical and nursing staff in perinatal care for individuals with disabilities necessary?

a) Νο

b) Yes

2. If Yes, do you consider the training/specialization of healthcare professionals in perinatal care for individuals with disabilities necessary at the level of:

a) Undergraduate

b) Postgraduate

c) Special seminars

d) All of the above

3. Do you believe that further training and specialization of medical and nursing staff in perinatal care for individuals with disabilities should be provided only to those who wish to specialize in this field?

a) No

b) Yes

Section 9: Data on the participants' knowledge of structures and services for providing perinatal care to individuals with disabilities

Please choose the correct answers.

Questions

1. Did you know if there are specialized centers/structures for providing perinatal care to individuals with disabilities in other countries?

a) No

b) Yes

2. Do you know if there are and what the structures in Greece for providing perinatal care to individuals with disabilities are?

a) No

b) Yes

If Yes, specify

3. Do you know the contact details of these structures?

a) No

b) Yes

4. Do you know which services you can turn to and get informed about the care of individuals with disabilities in Greece?

a) No

b) Yes

If Yes, specify

5. Do you know whom to contact within your workplace or a collaborating service, when, for example, you need to communicate via sign language with a patient with hearing problems?

a) No

b) Yes

If Yes, specify

Section 10: Attitudes of the participants toward providing perinatal care to women with disabilities

Please choose the correct answers.

Questions

1. Do you believe that women with disabilities need specialized parenting preparation courses?

a) No

b) Yes

2. Do you believe that a woman with disabilities should be accommodated in a different ward from women without disabilities?

a) No

b) Yes

3. Do you consider it beneficial to schedule separate appointments for pregnant women with disabilities on different days from women without disabilities?

a) No

b) Yes

4. Should these individuals be given priority in the provision of care?

a) No

b) Yes

5. Do you think a specialist is needed at the delivery of a woman with disabilities?

a) No

b) Yes

6. Do you believe that these women need psychological support during the perinatal period?

a) No

b) Yes

7. During the perinatal period, does a woman with disabilities need collaboration from multiple healthcare professionals?

a) No

b) Yes

Demographic and professional characteristics

The sample consisted of 149 midwives. One hundred and forty-six (98%) of the participants were females, with a mean age of 33.7 years (SD = 9.7 years), 60 (40.3%) were married, and 78 (52.3%) had a bachelor’s degree. The mean length of their work experience was 9.8 years (SD = 9.4 years), with 28 (18.8%) working in primary healthcare, specifically 17 (63%) of these in the public sector. Additionally, nine (23.7%) of those in secondary healthcare and 27 (45.8%) of those in tertiary healthcare work in the public sector. Finally, 48 (32.2%) report that their workplace was equipped for the perinatal care of individuals with disabilities. The demographic and professional characteristics are presented in Table 6.

**Table 6:** Demographic and professional characteristics of participants

Characteristic	N (%)
Gender	
Male	3 (2%)
Female	146 (98%)
Age (years) Mean (SD)	33.7 (9.7)
Marital Status	N (%)
Single	80 (53.7%)
Married	60 (40.3%)
Civil Partnership	4 (2.7%)
Divorced	5 (3.4%)
Education	N (%)
Midwifery Schools	7 (4.7%)
Bachelor’s degree	78 (52.3%)
Master’s degree (MSc)	62 (41.6%)
Doctorate (PhD)	2 (1.3%)
Years of Service Mean (SD) Median (Range)	98 (94), 6 (2-15)
Workplace & Employment	N (%)
Primary Health Care	28 (18.8%)
Secondary Health Care	38 (25.5%)
Tertiary Health Care	59 (39.6%)
Self-employed	24 (16.1%)
Primary Health Care (Specify exactly where)	N (%)
Public	17 (63%)
Private Practice	9 (33.3%)
Non-Governmental Organization (NGO)	1 (3.7%)
Public Primary Health Care (Specify exactly where)	N (%)
Refugee and Migrant Reception Centers	1 (5.9%)
Aretaieio Hospital	1 (5.9%)
National Public Health Organization	3 (17.6%)
Health Center	8 (47.1%)
Refugee Reception and Identification Center	1 (5.9%)
Athens Naval Hospital	1 (5.9%)
Alexandra Hospital	1 (5.9%)
SPMP & E	1 (5.9%)
Secondary Health Care (Specify exactly where)	N (%)
Public	9 (23.7%)
Private Clinic	29 (76.3%)
Tertiary Health Care (Specify exactly where)	N (%)
Public	27 (45.8%)
Private Hospital	32 (54.2%)
Does your workplace have equipment for the perinatal care of disabled individuals?	N (%)
No	101 (67.8%)
Yes	48 (32.2%)

Evaluation of maternity care services in Greece

Eighty-seven (58.4%) participants rated the maternity care services in Greece for individuals with disabilities as moderate, while the majority, 148 (99.3%), believe that the knowledge and specialization of the medical and nursing staff regarding perinatal care for individuals with disabilities are insufficient. On a personal level, 23 (15.4%) believe they have good knowledge regarding perinatal care issues for WWD. Additionally, 61 (40.9%) participants feel they have enough knowledge to use the correct terminology and words that do not contain elements of stigma and discrimination. The evaluation of midwife care services for WWD is displayed in Table 7.

**Table 7:** Evaluation of midwife care services for women with disabilities

Question		Ν	%
How do you evaluate the maternity care services in Greece for individuals with disabilities?	Not good at all	46	30.9
	Average	87	58.4
	Good	14	9.4
	Very Good	2	1.3
	Excellent	0	0
Do you consider that the knowledge and specialization of the medical and nursing staff on perinatal care for individuals with disabilities are sufficient?	No	148	99.3
	Yes	1	0.7
How do you evaluate your level of knowledge regarding perinatal care issues for women with disabilities?	I don't know	30	20.1
	I know moderately	86	57.7
	I know well	23	15.4
	I know very well	8	5.4
	I know excellently	2	1.3
Do you feel that you have enough knowledge to use the correct terminology and words that will not contain elements of stigma and discrimination?	No	88	59.1
	Yes	61	40.9

Emotional responses and attitudes

One hundred and nineteen (79.9%) feel comfortable gathering information from WWD about the nature of their disability. Sixty-eight (45.6%) of the participants stated that one of the emotions they experience when providing perinatal care to WWD is fear/anxiety, and 42 (28.2%) feel confidence/trust, while 117 (78.5%) believe that one of the things to avoid is showing pity/sympathy. Additionally, 41 (27.5%) believe that the most common emotion experienced by WWD during their perinatal care is concern about whether they will manage to carry the pregnancy to term (Table 8).

**Table 8:** Data pertaining to the emotions experienced by the participants during the provision of perinatal care to women with disabilities, as well as the emotions that participants believe that the women themselves experience during their perinatal care.

Question	Response	N	%
Do you feel comfortable gathering information from a woman about the nature of her disability?	No	30	20.1
	Yes	119	79.9
What are your feelings when providing perinatal care to women with disabilities?	Fear/anxiety	68	45.6
	Uncertainty, avoidance, and referral to another healthcare professional	10	6.7
	Neutral/indifferent	14	9.4
	Emotional burden (pity, sympathy)	41	27.5
	Confidence/trust	42	28.2
	Rejection	1	0.7
Which of the following do you consider should be avoided in the care of these women?	Expressing your personal opinion on matters concerning them	49	32.9
	Showing them pity/sympathy	117	78.5
	Not giving them the opportunity to decide for themselves and participate in their care, as well as in the care of their newborn	86	57.7
Which of the following, in your opinion, is the most common emotion experienced by women with disabilities during their perinatal care?	Fear for the health of their child	24	16.1
	Concern about whether they will manage to carry the pregnancy to term	41	27.5
	Fear of being separated from their child	5	3.4
	Insecurity about the quality of care they receive due to the lack of knowledge and specialization of healthcare professionals regarding their specific problem	29	19.5
	Insecurity about the adequacy and quality of parental care they will provide to their child themselves	14	9.4
	Feeling of loss of control	18	12.1
	Anxiety that the healthcare professional disapproves of their decision to be pregnant (stigma)	18	12.1

Views on reproductive rights and delivery methods

Table 9 presents the participants' views on the right of WWD to have children and the method of delivery they consider most appropriate depending on the case. Most participants, 142 (95.3%), believe in the right of WWD to have children, with 108 (72.5%) stating that the most appropriate method of delivery for a pregnant woman with mobility impairments (e.g., paraplegic) is a cesarean section and 119 (79.9%) stating that for a pregnant woman with sensory problems (e.g., vision, hearing), it is vaginal birth.

**Table 9:** Participants' views on the right of women with disabilities to have children and the method of delivery they consider most appropriate depending on the case.

Question		Ν	%
Do you believe in the right of women with disabilities to have children?	No	7	4.7
	Yes	142	95.3
What do you consider to be the most appropriate method of delivery for women with disabilities?			
For a pregnant woman with mobility impairments (e.g., paraplegic)	Vaginal birth	41	27.5
	Cesarean section	108	72.5
For a pregnant woman with intellectual disabilities (e.g., Down syndrome)	Vaginal birth	86	57.7
	Cesarean section	63	42.3
For a pregnant woman with psychiatric problems (e.g., schizophrenia)	Vaginal birth	57	38.3
	Cesarean section	92	61.7
For a pregnant woman with sensory problems (e.g., vision, hearing)	Vaginal birth	119	79.9
	Cesarean section	30	20.1

Experience and contact with WWD

Table 10 summarizes the experience and contact with WWD. Seventy-nine (53%) respondents have performed professional duties for WWD, with a mean occurrence of eight times (SD=16.6) and a median of three times (range 2-6). In the last year, this occurred 1.4 times on average (SD=1.9) with a median of one (range 0-2). Services were provided during pregnancy by 29 (36.7%), during childbirth by 38 (48.1%), and during postpartum by 57 (72.2%). When addressing a WWD during prenatal check-ups, 56 (37.6%) respondents addressed the woman first, three (2%) addressed her companion, 45 (30.2%) addressed both simultaneously, and 45 (30.2%) chose based on the situation. A specialized care plan for WWD was designed by 109 (73.2%) of the respondents. Personal experience with WWD varied, with 81 (56.6%) rarely having contact and only nine (6.3%) having daily interactions. Regarding personal or familial disability, 46 (30.9%) had a relative with a disability, while 50 (33.6%) had no personal or familial disability.

**Table 10:** . Experience/contact with women with disabilities.

Question	N	%
During your work, have you performed your professional duties for a woman with disability?		
No	70	47
Yes	79	53
If yes, how many times did it happen? Mean (SD), Median (range)	8 (16.6)	3 (2-6)
Specifically, how many times in the last year? Mean (SD), Median (range)	1.4 (1.9)	1 (0-2)
If yes, during which of the following periods of perinatal care did you provide services to a woman with disability?		
Pregnancy	29	36.7
Childbirth	38	48.1
Postpartum	57	72.2
During the prenatal check-up, a pregnant woman with disability and her companion arrive. Do you decide whom you will address first?		
The woman	56	37.6
Her companion	3	2
Both simultaneously	45	30.2
Depending on the situation	45	30.2
Do you and the rest of the team design a specialized care plan for a woman with disability?		
No	40	26.8
Yes	109	73.2
Indicate your personal experience or contact with a woman with disability including professional contact (e.g., colleague, person living in the same building, etc.); how frequent is it?		
Never	14	9.8
Rarely	81	56.6
Less than once a month	29	20.3
Weekly	10	7
Daily	9	6.3
Do you or someone in your family have a disability?		
Yes, I have a disability	3	2
Yes, a relative of mine has a disability	46	30.9
No, I do not have a disability	14	9.4
No, no relative of mine has a disability	36	24.2
No, I do not have a disability, and no relative of mine has a disability	50	33.6

Knowledge of WWD and perinatal care

Table 11 presents the responses of the participants regarding general knowledge about WWD and their perinatal care. In terms of correct answers, 32 (21.5%) participants responded that all the aforementioned categories of impairments belong to people with disabilities, 31 (20.8%) responded that 10-20% of the Greek population belongs to individuals with disabilities, 39 (26.2%) do not believe that WWD require more frequent perinatal care, 72 (48.3%) do not consider the presence of a neonatologist mandatory/necessary at the birth of a WWD, and 87 (58.4%) do not consider a pregnant WWD to be a high-risk pregnancy. The mean knowledge score was 35 points (SD = 24.5 points), while 28 (18.8%) participants scored zero, and 4 (2.7%) participants answered all questions correctly. Cronbach's α reliability coefficient for knowledge questionnaire was 0.71. Cronbach's α values of 0.7 or higher indicate acceptable internal consistency.

**Table 11:** Knowledge of the participants about individuals with disabilities and their perinatal care.

Question	N	%
Which of the following categories do you believe belong to people with disabilities?		
Mobility impairments	112	75.2
Sensory impairments	88	59.1
Intellectual impairments	99	66.4
Psychiatric impairments	52	34.9
Chronic conditions	0	0
All of the above	32	21.5
What do you believe is the percentage of the Greek population that belongs to individuals with disabilities?	N	%
˂5%	34	22.8
5-10%	84	56.4
10-20%	31	20.8
Do you believe that women with disabilities require more frequent perinatal care?	N	%
No	39	26.2
Yes	110	73.8
Do you consider the presence of a neonatologist mandatory/necessary at the birth of a woman with disabilities?	N	%
No	72	48.3
Yes	77	51.7
Do you consider a pregnant woman with disabilities to be a high-risk pregnancy?	N	%
No	87	58.4
Yes	62	41.6

Barriers to perinatal care for WWD

Table 12 presents the factors that constitute potential barriers to better perinatal care for WWD. The greatest barriers to better perinatal care for WWD, according to the participants, are the lack of services specifically adapted to their needs (148 participants (99.3%)) and the lack of infrastructure and availability of equipment at maternity hospitals and examination centers (143 participants (96%)). The behavior and experience of healthcare professionals regarding perinatal care for individuals with disabilities are perceived by participants as smaller barriers, (106 participants (71.1%) and 107 participants (71.8%), respectively). Barriers' numbers were summed up and a score was computed that could range from 0 to 9. The mean score was 7.6 (SD=1.6) and its reliability was acceptable (Cronbach’s α=0.76).

**Table 12:** Factors that constitute potential barriers to better perinatal care for women with disabilities.

Do you believe that the following constitute barriers to better perinatal care for women with disabilities?		N	%
The knowledge of healthcare professionals regarding perinatal care for individuals with disabilities	No	38	25.5
	Yes	111	74.5
The experience of healthcare professionals regarding perinatal care for individuals with disabilities	No	42	28.2
	Yes	107	71.8
Accessibility issues and physical barriers at maternity hospitals and examination centers for individuals with disabilities	No	16	10.7
	Yes	133	89.3
The lack of or difficult transportation for individuals with disabilities to maternity hospitals and examination centers	No	13	8.7
	Yes	136	91.3
The lack of infrastructure and availability of equipment at maternity hospitals and examination centers (e.g., mammography equipment that does not require the person to stand)	No	6	4
	Yes	143	96
Communication barriers between healthcare professionals and individuals with disabilities (e.g., professionals knowledgeable in sign language)	No	11	7.4
	Yes	138	92.6
The lack of services specifically adapted to the needs of individuals with disabilities	No	1	0.7
	Yes	148	99.3
The behavior of healthcare professionals toward individuals with disabilities	No	43	28.9
	Yes	106	71.1
Stereotypes and stigmatization of individuals with disabilities by healthcare professionals	No	38	25.5
	Yes	111	74.5

Need for education on perinatal care for WWD

The need for education on perinatal care for WWD is presented in Table 13. One hundred and forty-four (96.6%) participants stated that they consider additional training, specialization, and lifelong learning for all medical and nursing staff in perinatal care for WWD necessary, with 29 (20.1%) considering additional training at the undergraduate level necessary. Additionally, 24 (16.1%) believe that further training and specialization of medical and nursing staff in perinatal care for individuals with disabilities should be provided only to those who wish to specialize in this field. A score for the need for further training in perinatal care for individuals with disabilities was created. The score for the need for further training in perinatal care for individuals with disabilities ranged from 0 to 2 points, with a mean value of 1.8 points (SD = 0.4 points). Cronbach's α reliability coefficient was 0.72.

**Table 13:** Need for education on perinatal care for women with disabilities.

Question	N	%
Do you consider additional training, specialization, and lifelong learning for ALL medical and nursing staff in perinatal care for individuals with disabilities necessary?		
Νο	5	3.4
Yes	144	96.6
If yes, do you consider the training/specialization of healthcare professionals in perinatal care for individuals with disabilities necessary at the level of:	N	%
Undergraduate	29	20.1
Postgraduate	3	2.1
Special seminars	41	28.5
All of the above	71	49.3
Do you believe that further training and specialization of medical and nursing staff in perinatal care for individuals with disabilities should be provided only to those who wish to specialize in this field?	N	%
No	125	83.9
Yes	24	16.1

Knowledge of structures and services

Table 14 presents data on the participants' knowledge of structures and services for providing perinatal care to individuals with disabilities. Sixty-two (41.6%) participants stated that they know whether there are specialized centers/structures for providing perinatal care to individuals with disabilities in other countries, while only seven (4.7%) stated that they know whether there are corresponding structures in Greece and what they are, as well as the contact details for these. Additionally, 24 (16.1%) know which services they can turn to and get informed about the care of individuals with disabilities in Greece, and 39 (26.2%) know whom to contact within their workplace or a collaborating service when, for example, they need to communicate via sign language with a patient with hearing problems.

**Table 14:** Data on the participants' knowledge of structures and services for providing perinatal care to individuals with disabilities.

Question		Ν	%
Did you know if there are specialized centers/structures for providing perinatal care to individuals with disabilities in other countries?	No	87	58.4
	Yes	62	41.6
Do you know if there are Greece for providing perinatal care to individuals with disabilities and what they are?	No	142	95.3
	Yes	7	4.7
If Yes, specify	CARE	1	0.7
	I don't remember their names	1	0.7
	There are no specialized centers yet, just the institutions where perinatal care is provided	1	0.7
	Elena Venizelou	1	0.7
	KEA/AMEA	1	0.7
	NOISI, you can find such institutions nationwide	1	0.7
Do you know the contact details of these structures?	No	142	95.3
	Yes	7	4.7
Do you know which services you can turn to and get informed about the care of individuals with disabilities in Greece?	No	125	83.9
	Yes	24	16.1
If Yes, specify	Special centers, KEA-AMEA	1	0.7
	Special association of individuals with disabilities	1	0.7
	Daycare centers for individuals with disabilities	1	0.7
	Social service, centers for individuals with disabilities, NGOs	1	0.7
	Each organization addresses different types of disabilities many times	1	0.7
	Association of individuals with disabilities	1	0.7
Do you know whom to contact within your workplace or a collaborating service, when, for example, you need to communicate via sign language with a patient with hearing problems?	No	110	73.8
	Yes	39	26.2
If Yes, specify	General supervisor	2	1.3
	External collaborators	1	0.7
	Occupational therapist, psychologist, midwife	1	0.7
	Social worker	1	0.7
	Supervisor	2	1.3
	Special education service	1	0.7
	Psychologist, occupational therapist, social worker	1	0.7
	Psychologist, social worker, specialized doctor for the health problem	1	0.7

A score for knowledge of structures and services for providing perinatal care to individuals with disabilities was created. Scores range from 0 to 5 points, with higher scores indicating more knowledge of these structures. The knowledge score for structures and services for providing perinatal care to individuals with disabilities ranged from 0 to 5 points, with a mean value of 0.9 points (SD = 1.1 points). Cronbach's α reliability coefficient was 0.74.

Attitudes toward WWD perinatal care

Table 15 presents the attitudes of the participants toward providing perinatal care to WWD. One hundred and thirty-three (89.3) participants believe that priority should be given to these individuals in the provision of care, 124 (83.2%) believe that WWD need specialized parenting preparation courses, 118 (79.2%) believe that these women need psychological support during the perinatal period, 114 (76.5%) believe that during the perinatal period, collaboration from multiple healthcare professionals is necessary, and 68 (45.6%) believe that a specialist is needed at the delivery of a woman with disabilities. Additionally, 53 (35.6%) believe that a WWD should be accommodated in a different ward from women without disabilities, and 34 (22.8%) consider it beneficial to schedule separate appointments for pregnant WWD on different days.

**Table 15:** Attitudes of the participants toward providing perinatal care to women with disabilities.

Question		N	%
Do you believe that women with disabilities need specialized parenting preparation courses?	No	25	16.8
	Yes	124	83.2
Do you believe that a woman with disabilities should be accommodated in a different ward from women without disabilities?	No	96	64.4
	Yes	53	35.6
Do you consider it beneficial to schedule separate appointments for pregnant women with disabilities on different days from women without disabilities?	No	115	77.2
	Yes	34	22.8
Should these individuals be given priority in the provision of care?	No	16	10.7
	Yes	133	89.3
Do you think a specialist is needed at the delivery of a woman with disabilities?	No	81	54.4
	Yes	68	45.6
Do you believe that these women need psychological support during the perinatal period?	No	31	20.8
	Yes	118	79.2
During the perinatal period, does a woman with disabilities need collaboration from multiple healthcare professionals?	No	35	23.5
	Yes	114	76.5

A score for attitudes toward providing perinatal care to WWD was created. Scores range from 0 to 7 points, with higher scores indicating a more specialized attitude towards providing perinatal care to WWD. The score for attitudes toward providing perinatal care to WWD ranged from 1 to 7 points, with a mean value of 4.3 points (SD = 1.6 points). Cronbach's α reliability coefficient was 0.7.

## References

[1] (2011). World report on disability. Lancet.

[2] Byrnes L, Hickey M (2016). Perinatal care for women with disabilities: clinical considerations. J Nurse Pract.

[3] Matin BK, Williamson HJ, Karyani AK, Rezaei S, Soofi M, Soltani S (2021). Barriers in access to healthcare for women with disabilities: a systematic review in qualitative studies. BMC Womens Health.

[4] Mitra M, Akobirshoev I, Moring NS, Long-Bellil L, Smeltzer SC, Smith LD, Iezzoni LI (2017). Access to and satisfaction with prenatal care among pregnant women with physical disabilities: findings from a national survey. J Womens Health (Larchmt).

[5] Höglund B, Larsson M (2015). Midwives' comprehension of care for women with intellectual disability during pregnancy and childbirth: an open-ended questionnaire study in Sweden. Women Birth.

[6] Castell E, Stenfert Kroese B (2016). Midwives׳ experiences of caring for women with learning disabilities - a qualitative study. Midwifery.

[7] Signore C, Spong CY, Krotoski D, Shinowara NL, Blackwell SC (2011). Pregnancy in women with physical disabilities. Obstet Gynecol.

[8] Mitra M, Smith LD, Smeltzer SC, Long-Bellil LM, Sammet Moring N, Iezzoni LI (2017). Barriers to providing maternity care to women with physical disabilities: perspectives from health care practitioners. Disabil Health J.

[9] Sarantaki A, Zambeli MV, Diamanti A, Lykeridou A (2021). Health care providers’ knowledge and attitudes in perinatal care for women with disabilities. J Med Clin Res Rev.

[10] Temane AM, Magagula FN, Nolte AG (2024). Midwives' lived experiences of caring for women with mobility disabilities during pregnancy, labour and puerperium in Eswatini: a qualitative study. BMC Womens Health.

[11] Magagula F, Temane A, Nolte AG (2022). Women with mobility disabilities' experiences of maternity care during pregnancy, labour and puerperium in Eswatini. Health SA.

[12] Redshaw M, Malouf R, Gao H, Gray R (2013). Women with disability: the experience of maternity care during pregnancy, labour and birth and the postnatal period. BMC Pregnancy Childbirth.

[13] König-Bachmann M, Zenzmaier C, Schildberger B (2019). Health professionals' views on maternity care for women with physical disabilities: a qualitative study. BMC Health Serv Res.

[14] Devkota HR, Murray E, Kett M, Groce N (2017). Healthcare provider's attitude towards disability and experience of women with disabilities in the use of maternal healthcare service in rural Nepal. Reprod Health.

[15] Smeltzer SC, Mitra M, Long-Bellil L, Iezzoni LI, Smith LD (2018). Obstetric clinicians' experiences and educational preparation for caring for pregnant women with physical disabilities: a qualitative study. Disabil Health J.

[16] Ekstrand Ragnar M, Niemeyer Hultstrand J, Tydén T, Larsson M (2018). Development of an evidence-based website on preconception health. Ups J Med Sci.

[17] Hristova-Atanasova E, Iskrov G, Raycheva R, Mandova V, Stefanov R (2023). Preconception-health-related attitudes of Bulgarian women of reproductive age. Healthcare (Basel).

[18] Cinelli G, Croci I, Gesualdo F, Pandolfi E, Miller KP, Tozzi AE (2023). Public engagement in digital recommendations for promoting healthy parental behaviours from preconception through the first 1000 days. Int J Environ Res Public Health.

